# Toward a navigation framework for fetoscopy

**DOI:** 10.1007/s11548-023-02974-3

**Published:** 2023-08-16

**Authors:** Alessandro Casella, Chiara Lena, Sara Moccia, Dario Paladini, Elena De Momi, Leonardo S. Mattos

**Affiliations:** 1 Department of Advanced Robotics, Istituto Italiano di Tecnologia, Genoa, Italy; 2 Department of Electronic, Information and Bioengineering, Politecnico di Milano, Milan, Italy; 3 Department of Excellence in Robotics and AI, The BioRobotics Institute, Scuola Superiore Sant’Anna, Pisa, Italy; 4 Department of Fetal and Perinatal Medicine, Istituto Giannina Gaslini, Genoa, Italy

**Keywords:** Fetal surgery, Mosaicking, Occlusion recovery, Twin-to-twin transfusion syndrome, Fetoscopy

## Abstract

**Purpose:**

Fetoscopic laser photocoagulation of placental anastomoses is the most effective treatment for twin-to-twin transfusion syndrome (TTTS). A robust mosaic of placenta and its vascular network could support surgeons’ exploration of the placenta by enlarging the fetoscope field-of-view. In this work, we propose a learning-based framework for field-of-view expansion from intra-operative video frames.

**Methods:**

While current state of the art for fetoscopic mosaicking builds upon the registration of anatomical landmarks which may not always be visible, our framework relies on learning-based features and keypoints, as well as robust transformer-based image-feature matching, without requiring any anatomical priors. We further address the problem of occlusion recovery and frame relocalization, relying on the computed features and their descriptors.

**Results:**

Experiments were conducted on 10 in-vivo TTTS videos from two different fetal surgery centers. The proposed framework was compared with several state-of-the-art approaches, achieving higher $\textrm{SSIM}_{5}$ on 7 out of 10 videos and a success rate of $93.25\%$ in occlusion recovery.

**Conclusion:**

This work introduces a learning-based framework for placental mosaicking with occlusion recovery from intra-operative videos using a keypoint-based strategy and features. The proposed framework can compute the placental panorama and recover even in case of camera tracking loss where other methods fail. The results suggest that the proposed framework has large potential to pave the way to creating a surgical navigation system for TTTS by providing robust field-of-view expansion.

**Supplementary Information:**

The online version contains supplementary material available at 10.1007/s11548-023-02974-3.

## Introduction

Twin-to-twin transfusion syndrome (TTTS) is a rare complication affecting 10–15% of monochorionic diamniotic pregnancies where twins are affected by unbalanced and chronic blood transfer through placental anastomoses [1] treated with selective laser photocoagulation [2] performed in fetoscopy. The procedure is particularly challenging due to the limited field-of-view (FoV), poor visibility due to amniotic fluid turbidity, and variability in illumination that can negatively impact on surgery duration and lead to residual anastomoses, resulting in persistent TTTS. In the context of fetoscopy for TTTS, surgical data science (SDS) methodologies have been exploited to provide surgeons with context awareness and decision support with anatomical structure segmentation [3–7] and mosaicking.

Most of the work in the literature for mosaicking focuses on handcrafted features or requires accurate anatomical structure segmentation which can compromise registration robustness [8]. To tackle this problem, an alternative solution is to rely on stable keypoints. Previous work highlighted that classical algorithms for keypoint detection (i.e., SIFT, ORB) cannot tackle the challenges of intra-operative fetoscopic images [9]. Exploiting learning-based methods for detecting keypoints can be a solution.

Furthermore, in fetoscopy videos many events (e.g., fetal movements, maternal pulses, loss of focus) could compromise the frame tracking hampering mosaicking reconstruction. While this problem has not been addressed in fetoscopy, in closer fields researchers are exploring simultaneous localization and mapping (SLAM) approaches [10–12].

On this basis, in this work we propose an integrated learning-based navigation framework for frame registration from intra-operative fetoscopic videos that can provide robust mosaicking with occlusion recovery. We can summarize our contributions as follows:

- We propose a framework inspired by Visual SLAM [13] which does not rely on any anatomical priors for mosaicking.
- We show that keypoints and features extracted with the proposed framework pretrained on non-medical data can tackle the lack of annotated data for feature extraction in fetoscopy.
- We experimentally validate our approach on 10 in-vivo TTTS video sequences, 4 provided by Istituto Giannina Gaslini (Genoa, Italy), and 6 from the extended version of the dataset presented in [9] for fair comparison with the literature.

### Related work

In the last years, mosaicking has been investigated with the purpose of supporting fetal surgeons by providing FoV expansion. The first attempts to obtain panoramic placental images were based on traditional keypoints extracted from fetoscopic images and matched to estimate the relative transformations, as described in [8]. Currently, researchers are exploring deep-learning strategies. In [14], a convolutional neural network (CNN) is trained to detect stable image regions around large veins, and the corners of their bounding boxes are aligned to achieve registration. Despite the promising results, CNN is trained with phantom images which cannot encode properly the real challenges of intra-operative images.

The work in [15] proposed a CNN trained with controlled data augmentation for pairwise homography estimation. However, texture paucity and the high image variability make homography estimation challenging and this may translate in drift or even failure of mosaicking.

The work in [9] relied on vessel segmentation map from consecutive frames obtained by a CNN and then registered by Lucas–Kanade (LK) algorithm. Registration performance is high when vessels are clearly visible. However, vessels can be challenging to see or non-visible at all. Furthermore, the time required for frame registration is unsuitable for real-time applications.

More recently, [16] proposes a method based on optical flow for homography estimation and thus not requiring vessel map for mosaicking. However, optical flow assumes brightness constancy and strong texture [17], which cannot be always guaranteed in fetoscopic frames.

Despite several methods for mosaicking have been proposed, few work has been done to tackle occlusion recovery and frame relocalization in TTTS. The work in [18] proposed an offline occlusion recovery based on the cosine distance between VGG16 features of each frame. Despite the promising results, the computational time to process a frame pair is not compatible with real-time application.

Researchers in close fields are exploiting SLAM for endoscopy, introducing the use of CNN for extracting features. The use of learned features was shown to provide better mapping and relocalization accuracy [19]. Although the wide variety of applications of SLAM techniques, its use in endoscopy is still very limited and a full navigation framework for fetoscopy has not been explored yet. Therefore, our aim is to investigate if learning-based keypoints and features can tackle fetoscopic images challenges and thus lay the foundation for a SLAM framework in fetoscopy to provide support for navigation during fetal surgery.

## Method

**Fig. 1:**
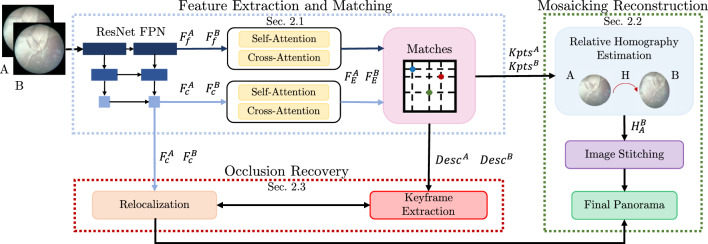
Overview of the proposed framework for fetoscopic images mosaicking. During the feature extraction phase, features from different pyramid levels are extracted from the input frames (*A*, *B*) and transformed by self-attention and cross-attention ($F_c^{A}$ and $F_c^{B}$). $F_{f}^{A}$ and $F_{f}^{B}$ indicate fine features, while $F_{c}^{A}$ and $F_{c}^{B}$ coarse features. A matching module performs coarse matches and a further refinement, producing keypoints ($\mathrm {{Kpts}^{A}}$ and $\mathrm {{Kpts}^{B}}$) and descriptors ($\mathrm {{Descr}^{A}}$ and $\mathrm {{Descr}^{B}}$). Descriptors are used for keyframe extraction, keypoints for homography estimation and then panorama reconstruction. Coarse features are necessary to perform recovery on the global panorama through a comparison with the keyframes found

The workflow of our proposed framework is shown in Fig. 1. The first block is the feature extraction and matching block, described in Sect. “Feature Extraction and Matching”, which processes pairs of consecutive frames (*A*, *B*) and outputs features ($F_{c}^{A}$, $F_{c}^{B}$), matching keypoints ($\textrm{Kpts}^A$, $\textrm{Kpts}^B$) and descriptors ($\textrm{Desc}^A$, $\textrm{Desc}^B$). The keypoints are used to estimate the homography ($H_{A}^{B}$) between *A* and *B* for mosaicking reconstruction, as described in Sect. “Mosaicking reconstruction”. Combining features and matching descriptors allows us to achieve occlusion recovery, as described in Sect. “Occlusion recovery”.

### Feature extraction and matching

The proposed method for feature extraction and matching is inspired on local feature matching with transformers (LoFTR) [20].

Multi-scale features ($F_c^{A}$, $F_c^{B}, F_f^{A}$, $F_f^{B}$) are extracted from ResNet FPN. $F_c^{A}$ and $F_c^{B}$ are features at coarser level that can be processed efficiently but lose spatial information; thus, prior positional encoding by the transformer module is performed.

Features from the transformer module ($F_E^{A}$, $F_E^{B}$) are matched using the confidence matrix ($P_c$) as:

1 $$\begin{aligned} P_c(i,j) = {\textrm{softmax}}(S (i, \cdot ))_j \times {\textrm{softmax}}(S (\cdot , i))_j \end{aligned}$$

where *i* and *j* are the $i-\text {th}$ and $j-\text {th}$ coarse matches, while *S* indicates the score matrix between the features:

2 $$\begin{aligned} S(i,j) = \frac{1}{\tau } \times \left\langle { {F}}_{E}^{A}(i), { {F}}_{E}^{B}(j)\right\rangle \end{aligned}$$

Since the score matrix is computed for coarse features the temperature ($$\tau $$) takes into account its uncertainty. The feature matching is then performed through mutual nearest neighbor ($$\text {MNN}$$). Matches ($$M_{c})$$ are identified as:

3 $$\begin{aligned} M_{c} = \{ (i,j) |\forall (i,j) \in \text {MNN}(P_{c}, P_{c}(i,j) \ge \theta _{c})\} \end{aligned}$$

where $P_c$ is the feature matching confidence matrix. Matches with confidence lower than a predefined threshold (${\theta _{c}}$) are discarded, to avoid noisy results due to incorrect matches. Different experiments were conducted to select the best threshold value. Low values of ${\theta _{c}}$ increase noisy matches, negatively impacting algorithm performances. High values of ${\theta _{c}}$ lead to the identification of a high number of close keyframes, slowing the computation. For this reason, the best value was identified as 50%.

Finally, the coarse-to-fine module performs a final refinement by computing the expectation over the probability distribution between encoded features at coarse and fine level.

**Fig. 2:**
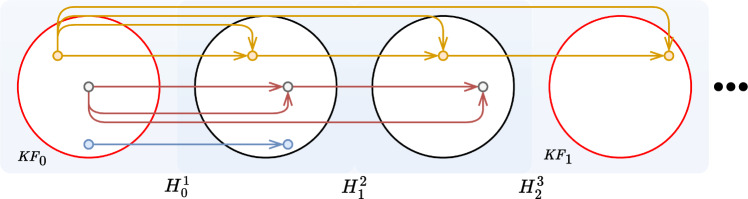
Graphical overview of the keyframe extraction algorithm, described in Sect. "Keyframes extraction". Keyframes are highlighted in red (*i.e.,* $\textrm{KF}_0, \textrm{KF}_1$), while the small colored dots are the keypoints, the arrows highlight keypoints matched between frames

### Mosaicking reconstruction

The set of matching keypoints computed with the method described in Sect. “Feature Extraction and Matching” is used to estimate the relative homography ($H_A^{B}$) between two consecutive frames through robust RANSAC. Each relative homography is then computed with respect to the global reference frame, *i.e.,* a blank canvas where the final mosaic is contained. Finally, each new frame is warped and post-processed with exposure fusion algorithm.

Due to the intrinsic characteristics of the placental environment and its relative position with respect to the camera, fetoscopic images are not homogeneous in illumination: the central part, directly hit by endoscopic light, is brighter, while toward the border the illumination level decreases. Thus, when images are stitched together, darker circular shadows can be seen in correspondence of the borders, worsening the visual quality of the reconstruction. In order to get uniform scene exposure and, as a result, softer shadows, we used an algorithm based on exposure blending by [21].

### Occlusion recovery

Due to the challenges associated with fetoscopic images, which are outlined in Sect. “Introduction”, keypoint tracking could be lost during placenta examination, leading to the failure of the mosaicking reconstruction. In order to address this issue, we design a recovery strategy which resumes the mosaic as soon as valid keypoints have been identified. The recovery algorithm is divided in two steps: (i) keyframes extraction (Sect. “Keyframes extraction”) and (ii) frame relocalization (Sect. “Relocalization”).

#### Keyframes extraction

The idea of keyframe extraction comes from the observation that in a video sequence, especially when camera movements are limited, close frames carry very similar semantic information. Thus, considering all the input frames for recovery would be redundant. A graphical schema of the following algorithm is shown in Fig. 2. In our method, the first frame of the sequence is considered as the first keyframe. From the first keyframe, we keep the descriptors ($\textrm{Descr}$), obtained as described in Sect. “Feature extraction and matching”, and compute the number of matched descriptors with the following frame. We continue this process until the number of matching descriptors falls below a threshold ($T_{\text {discard}}=10\%$), whose value is set experimentally. When this threshold is reached, the next frame is selected as a new keyframe. Descriptors matching, as for the mosaicking task (Sect.  “Mosaicking reconstruction”), is performed through a $\textrm{MNN}$ algorithm.

It could still happen that two frames really close in the sequence are both selected as keyframes. To avoid this redundancy, if the computed Euclidean distance between the features of consecutive keyframe-pair is lower than a threshold ($T_{\text {KF}}$, experimentally set at 1300), only the first added keyframe is kept, while the other is discarded.

#### Relocalization

The aim of the relocalization task is to correctly register a frame on the final mosaic by recovering the loss of tracking when the camera tracking fails. Relevant global features are extracted from each keyframe, and the Euclidean distance between all the keyframes and the frame to relocalize is computed. This procedure allows to identify the nearest keyframe candidates achieving a quick recovery compatible with clinical requirements. Matching keypoints are used to estimate the relative transformation to register the frame with respect to the mosaic already generated. Once the registration has been recovered, the mosaicking reconstruction described in Sect.  “Mosaicking reconstruction” restarts.

## Experimental protocol

The dataset used in this work is the combination of the extended fetoscopic dataset presented in [6] and a property dataset from Istituto Giannina Gaslini (Genoa, Italy). The dataset includes a total of 10 videos and 2344 frames. Examples of dataset frames are represented in the supplementary materials. This multi-center dataset allows us to develop robust solutions considering most challenges in intra-operative fetoscopic image analysis, such as turbidity of the amniotic fluid, high variability of illumination, occlusions, texture paucity and poor image quality.

In our experiments, we compared our framework to the state of the art for the two tasks, mosaicking and occlusion recovery, independently.

We compared our framework for feature extraction and matching (Sect. “Feature extraction and matching”) and mosaicking reconstruction (Sect. “Mosaicking reconstruction”) to Bano et al. [9], which is the most recent and similar work to ours, and with approaches based on classical keypoints:

- Experiment M1 (*EM1*): Bano et al. [9]
- Experiment M2 (*EM2*): SIFT+RANSAC [22, 23]
- Experiment M3 (*EM3*): ORB+RANSAC [24]
- Experiment M4 (*EM4*): Proposed framework

For fair comparison with [9], mosaicking performances were evaluated in terms of Structural Similarity Index Measure (SSIM) [25].

**Fig. 3:**
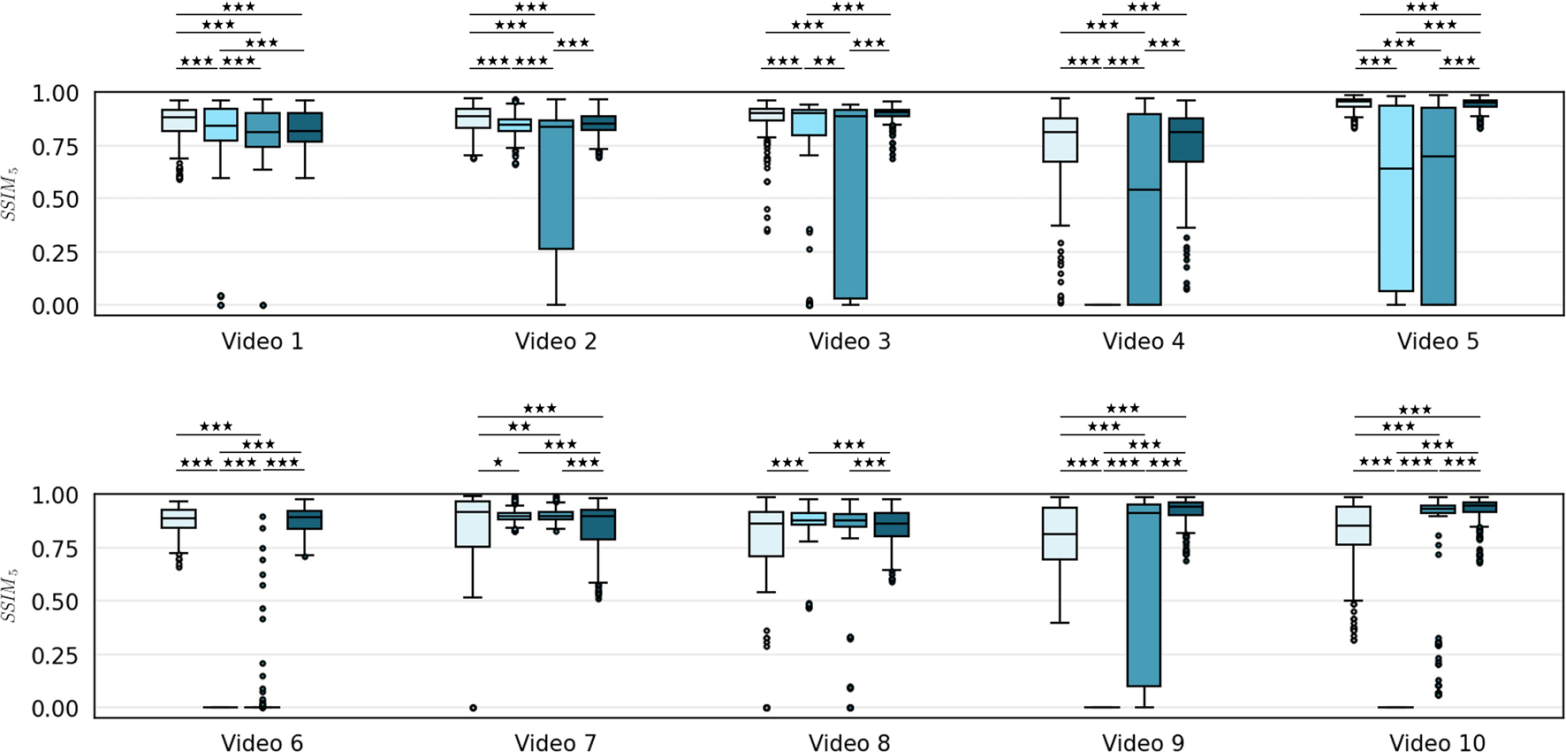
Performance comparison in terms of $$\textrm{SSIM}_5$$ between (in order from left to right) Bano et al. [9] (*EM1*), SIFT (*EM2*), ORB (*EM3*) and the proposed method (*EM4*). Wilcoxon statistical tests have been performed to assess statistical differences ($$^* p< 0.05, ^{**}p< 0.01, ^{***}p < 0.001$$)

According to the findings in Bano et al. [9], SSIM is almost constant in case of very small displacements as typically observed in fetoscopy. For this reason, using a 5-frame SSIM ($\textrm{SSIM}_5$) is better suitable for validation. Wilcoxon test was used to evaluate the statistical differences between the implemented methods.

We then compared our occlusion recovery strategy (Sect. “Occlusion recovery”) to:

- Experiment R1 (*ER1*): the recovery approach used in [18] using VGG16
- Experiment R2 (*ER2*): the recovery approach used in [18] using ResNet50
- Experiment R3 (*ER3*): SIFT with the occlusion recovery presented in [26]
- Experiment R4 (*ER4*): ORB with the occlusion recovery presented in [26]

From this set of experiments, we excluded Bano et al. [9] because it does not embed any recovery strategy. To assess the robustness of the recovery algorithm, different tests were performed. For every video sequence, a random frame was selected while ensuring it was not a keyframe. At this point, the algorithm described in Sect. “Occlusion recovery” was applied to relocalize the randomly extracted frame. The same procedure was applied to a transformed version of the same frame, which was performed to assess the robustness of the method applying corruptions and affine distortions to images. The recovery performance was evaluated using the *Success Rate* metric, defined as:

4 $$\begin{aligned} {\mathrm{Success~ Rate}} = \frac{\#{\mathrm{correct\,\, recoveries}}}{\#{\mathrm{total\,\, recoveries}}}\times 100 \end{aligned}$$

## Results and discussion

**Fig. 4:**
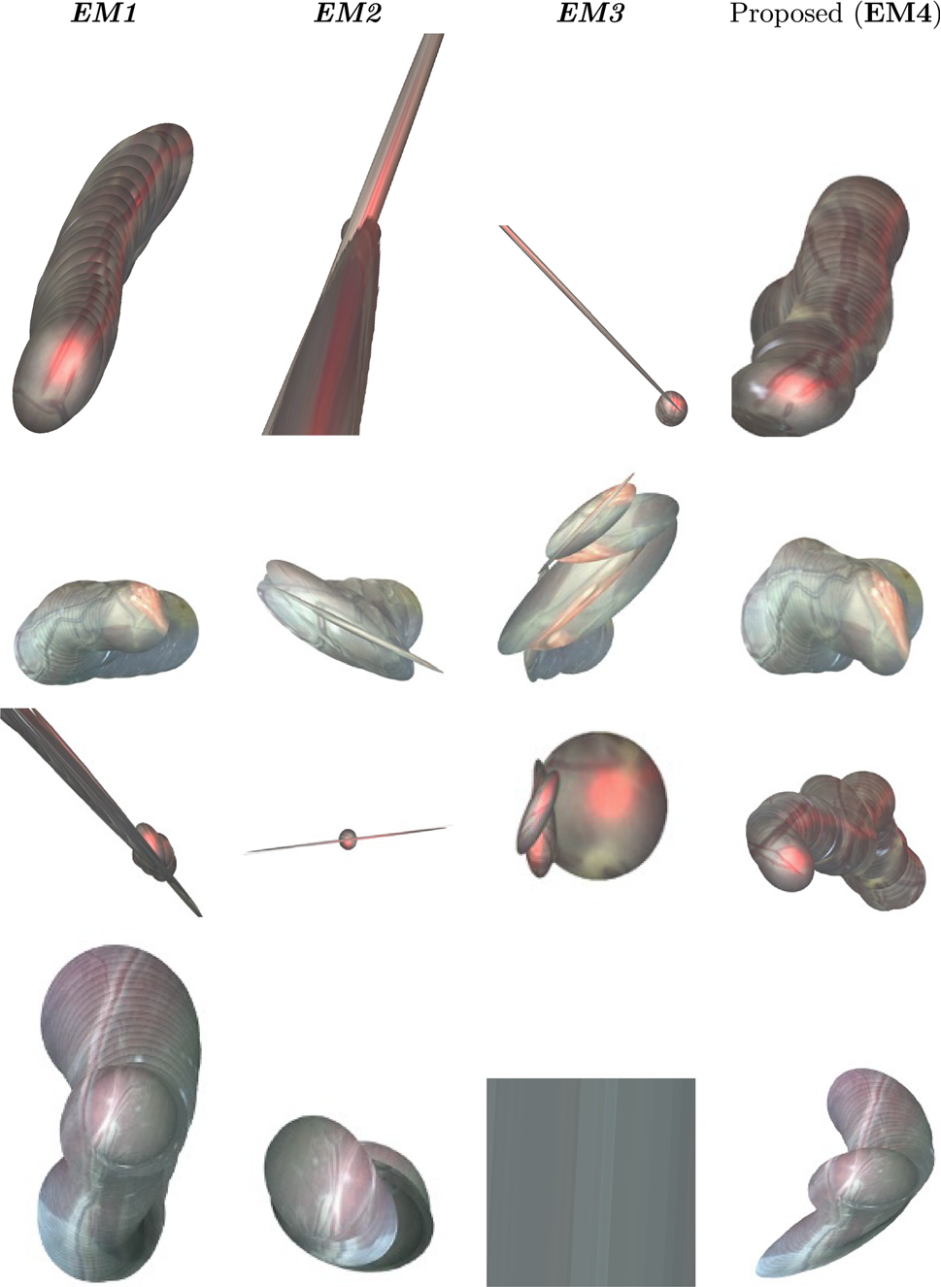
Mosaicking comparison between Bano et al. (*EM1*), SIFT (*EM2*), ORB (*EM3*) and the proposed method (*EM4*) on four dataset videos. Outcomes show a large variability between different methods and between different videos

Figure 1 illustrates the performance comparison of mosaicking reconstruction between the proposed method and the state of the art, with respect to Y. Meanwhile, Table 1 presents the average ($\overline{\textrm{SSIM}}_5$) and standard deviation for Y. Examples of the generated mosaics for all the tested methods can be seen in Fig. 4. From the boxplot in Fig. 3, it can be seen that the proposed framework (*EM4*) outperforms traditional approaches like SIFT (*EM2*) and ORB (*EM3*), and achieves comparable or superior performance in terms of $\textrm{SSIM}_5$ compared to Bano et al. [9] (*EM1*). Result of Wilcoxon test for $\textrm{SSIM}_5$ highlight the significant difference in performances of the proposed method compared to the other tested methods.

All the methods achieved good performance on Video 1, in particular *EM1* achieved the highest $\overline{\textrm{SSIM}_5}$ (0.8556), as expected, due to the good placental vessel visibility and negligible lens distortions. The proposed method achieved lower but still comparable results ($\overline{\textrm{SSIM}_5} = 0.8257$), while traditional methods (*EM2* and *EM3*) still struggled in computing the right homography, as highlighted by the outliers in the boxplot, resulting in important drift in the final mosaic.

In Video 2, vessels are well visible and *EM1* still achieved higher $\overline{\textrm{SSIM}_5}$ (0.8714) compared to *EM4* (0.8497), but the view in Video 2 is not planar for the entire sequence. Bano et al. tended to produce flat-looking mosaic, instead learning-based methods were able to deal with different orientations of the placenta providing better consistency in the final mosaic. *EM2* and *EM3*, as can be seen in Fig. 4, are not able to correctly generate the final mosaic. *EM2* strongly underestimated the homography, leading to the positioning of all the frames on top of the first one, which explains the low SD 0.0563. Conversely, the high SD of *EM3* (0.3585) suggests homography overestimation.

**Table 1:** Mosaicking performance in terms of mean ± standard deviation of $\textrm{SSIM}_5$ for all the videos in the dataset between Bano et al. (*EM1*), SIFT (*EM2*), ORB (*EM3*) and the proposed framework without occlusion recovery (*EM4*)

Video #	*EM1*	*EM2*	*EM3*	*EM4*
1	$\mathbf {0.8556 \pm 0.0809}$	${ 0.8241 }\pm { 0.1512}$	${ 0.8098 }\pm { 0.1170}$	$0.8257 \pm 0.0834$
2	$\mathbf {0.8714 \pm 0.0666}$	${ 0.8441 }\pm { 0.0563}$	$0.6353 \pm 0.3585$	$0.8497 \pm 0.0553$
3	$0.8670 \pm 0.1079$	${ 0.7658 }\pm { 0.3060}$	$0.5713 \pm 0.4197$	$\mathbf {0.8946 \pm 0.0440}$
4	$0.7457 \pm 0.1921$	–	$0.4526 \pm 0.4134$	$\mathbf {0.7526 \pm 0.1773}$
5	$\mathbf {0.9445 \pm 0.8720}$	$0.5415 \pm 0.4021$	$0.4911 \pm 0.4339$	$0.9406 \pm 0.0316$
6	$0.8720 \pm 0.0646$	–	$0.0309 \pm 0.1358$	$\mathbf {0.8750 \pm 0.0610}$
7	$0.8133 \pm 0.2429$	${ 0.8974} \pm { 0.0333}$	${ 0.8999 }\pm { 0.0295}$	$\mathbf {0.8455 \pm 0.1200}$
8	$0.7603 \pm 0.2623$	$0.8740 \pm 0.0710$	${ 0.7961 }\pm { 0.2560}$	$\mathbf {0.8463 \pm 0.0808}$
9	$0.7947 \pm 0.1506$	–	$0.5777 \pm 0.4129$	$\mathbf {0.9195 \pm 0.0598}$
10	$0.8155 \pm 0.1649$	–	${ 0.8209} \pm { 0.2749}$	$\mathbf {0.9239 \pm 0.0653}$

The highest average values are shown in bold. The values in italic represent mosaics that were discarded after visual inspection due to errors in reconstruction

From Video 3 to Video 6, *EM4* achieved comparable value of $\overline{\textrm{SSIM}_5}$ with *EM1*, while *EM2* and *EM3* failed. In particular, for *EM2* some boxplots are not shown because the algorithm failed on the entire sequence. Analyzing in details these results, *EM1* cannot keep continuous frame tracking thus corrupting the final mosaic. This can be explained due to poor visual conditions that hinder CNN vessel segmentation, such thin or absent vessels, non-planarity of the scene and major lens distortions. Furthermore in videos 7 to 10, the presence of the laser pointer and low illumination compromise visibility and texture quality negatively affecting classical descriptor-based methods as well, while $\textit{EM4}$ was able to successfully compute a decent mosaic.

The poor performances of traditional descriptor-based methods were in fact expected due to fetoscopic images challenges introduced in Sect. "Introduction". The use of learning-based keypoints in $\textit{EM4}$, on the other hand, was able to better address these challenges and provide more robust mosaicking capability. A graphical comparison of the described feature extraction and matching methods can be found in the supplementary materials. Tracking was never lost during our tests. However, some minor distortions are still present especially in presence of very fast movements, loss of focus or presence of noisy texture-less regions.

In addition to the limitations reported in Sect. “Related work”, the LK registration of $\textit{EM4}$ require around 1 s for image pair. Instead, $\textit{EM4}$ can process an average of 10 image pairs per second, with average performances of $17.26 \pm 3.01$ ms for feature extraction and $18.78 \pm 2.36$ ms on A100 40 GB GPU with 64 GB RAM and 8 CPUs. Even though the real-time requirements are not fulfilled yet, this can be considered a promising step in that direction.

The fetal environment is highly dynamic, as introduced in Sect. “Introduction”. An effective method for occlusion recovery and frame relocalization is needed to achieve robust mosaicking algorithm to support clinicians during the procedure. Furthermore, such framework can be used along to identify loop-closures for global optimization algorithms [27]. Qualitative results for the recovery task are shown in supplementary materials. *ER2* reaches the best outcome among feature based methods, with a success rate of $83.13\%$. The ResNet50 feature extraction from *ER2* resulted more effective than VGG from *ER1*, which obtained a recovery success rate of $26.25\%$.

Classical descriptors, like *ER3* ($\textit{Success Rate} = 20.00\%$) and *ER4*, suffered from large particles and illumination variability, failing to detect robust keypoints and strong descriptors to correctly relocalize a frame for both tasks. As a consequence, the number of keypoints and descriptors found by such methods is low, leading to high probability of mosaicking and relocalization failure. The learning-based descriptors used in the proposed method demonstrated to be also effective for occlusion recovery, achieving the highest $\textit{Success Rate}$ ($93.75\%$).

Inspecting the relocalization results (in supplementary materials), we can note that for Video 1 all methods successfully identified the keyframe from which the mosaic should be recovered. However, except for the proposed method, all the other methods were not able to correctly register the new frame with the keyframe.

In Video 8, *ER1* and *ER2* poorly performed, probably due to the constant presence of the laser which compromised the extraction of unique frame features for occlusion recovery. On the other hand, *ER4* and the proposed method detected multiple keyframe candidates. However, in this sequence, classical descriptors were not so robust, leading to wrong relocalizations.

In Video 10, the dim illumination and low visibility of vascular structures impacted descriptor-based methods. *ER3* couldn’t recover from occlusion and found no keyframe candidate, whereas *ER4* identified an incorrect keyframe. Among the feature-based methods, *ER1* also failed to correctly identify the right keyframe. However, both *ER2* and the proposed method accurately identified the closest keyframe.

Focusing on the results achieved by *ER3* and *ER4*, it is reasonable to conclude that their low recovery performances were caused by the descriptors characteristics, which were not suited to deal with fetoscopic data, confirming results from the literature [9]. VGG features can successfully handle occlusions and recovery when images are rich in texture but fail in case of challenging visual conditions where there are low or no textures. Not surprisingly more complex features extraction backbones, such as ResNet50, achieve overall better performance. However, ResNet50 can struggle in discriminating very similar keyframes or when disturbing factors are present such as the laser pointer. In the proposed framework, the use of learning-based descriptors can be seen as a hybrid method that combines the advantages of descriptors and features.

## Conclusion

This paper proposed a learning-based framework for placental mosaicking with occlusion recovery based solely on intra-operative videos. To the best of our knowledge, this is the first attempt to manage occlusions in a fetoscopic mosaicking pipeline. The proposed method follows state-of-the-art assumption of rigid scenes [9]. However, this assumption may not hold in all intra-operative videos, where maternal breathing, pulses, or fetal movements could cause drift accumulation in the registration. In order to address this challenge, future works should exploit the problem of deformable registration. The results achieved suggest that this new framework is able to reliably reconstruct the placental panorama even when the tracking from fetoscopic camera is lost, as the recovery task allows to relocalize the frame in correspondence of which the stitching algorithm failed. This is a promising solution to assist surgeons during TTTS surgery, which currently suffers with the issue of very limited FoV. A broader view of the placenta could decrease the duration of the surgical intervention by facilitating the identification of pathological anastomoses and the verification of their proper treatment.

### Supplementary Information

Below is the link to the electronic supplementary material.

Supplementary file 1 (pdf 21297 KB)
