# Supplementary material for: Toward a navigation framework for fetoscopy
Source: Int J Comput Assist Radiol Surg. 2023 Aug 16;18(12):2349–56. doi: 10.1007/s11548-023-02974-3 (PMC10632301; doi:10.1007/s11548-023-02974-3)
Supplement: Supplementary file 1 — (pdf 21297 KB) [file 11548_2023_2974_MOESM1_ESM.pdf]

Supplementary Materials

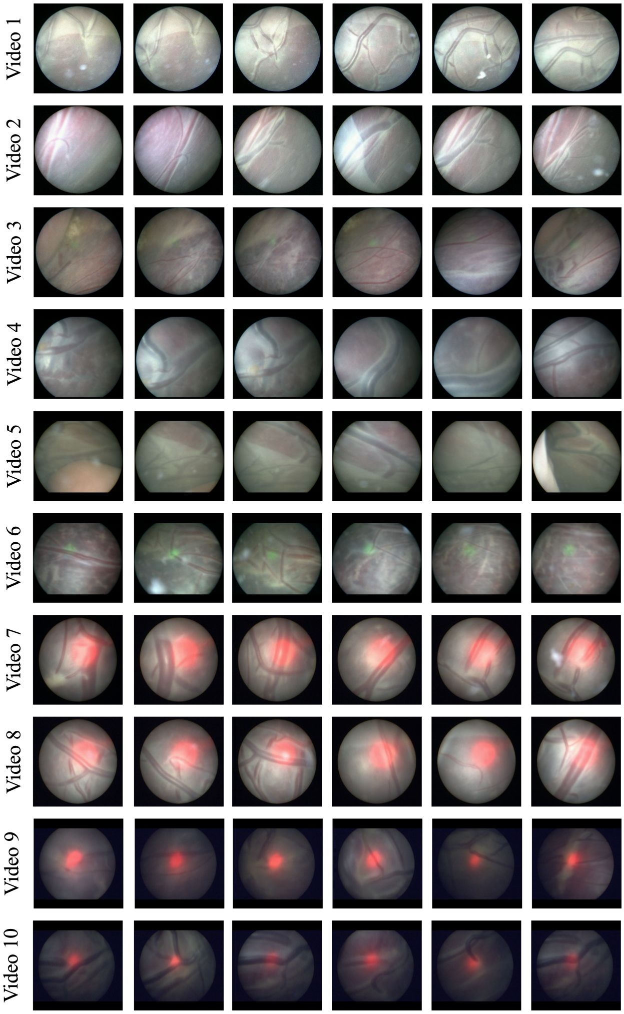

Figure 1: Examples of frames from the described dataset, for each sequence six frames are shown.

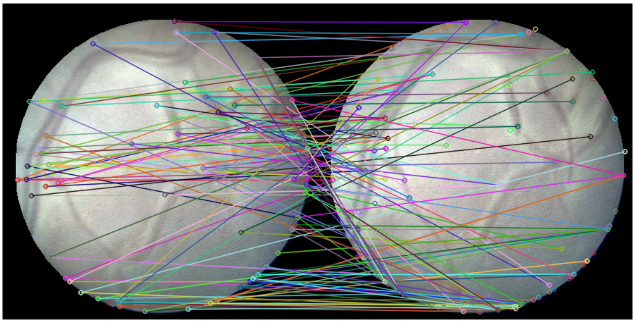

(a)

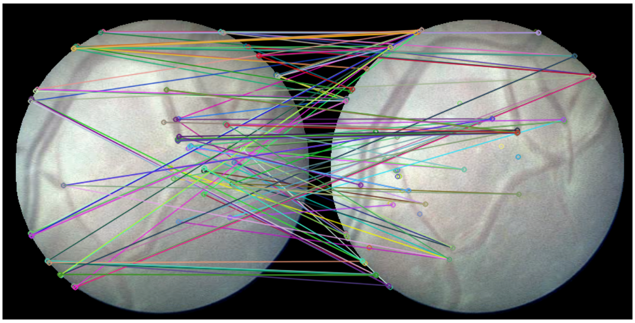

(b)

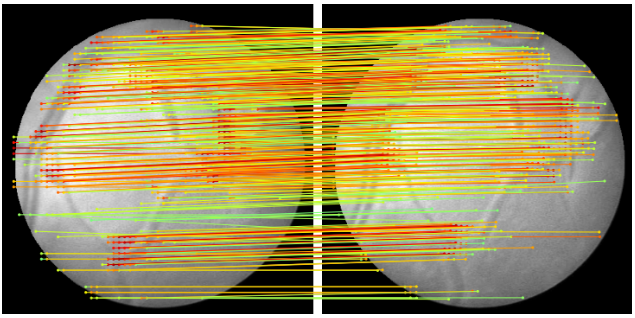

(c)

Figure 2: Graphical comparison between matches found by the three considered algorithms for keypoints extraction and matching in two consecutive frames. Keypoints are selected and matched by SIFT in Fig. (a), ORB in Fig. (b), LoFTR in Fig. (c). From the given example a significant difference in the number of detected keypoints between the algorithms is clear: 73 keypoints are found by SIFT, 79 by ORB, 523 by LoFTR (just the keypoints with a confidence level greater than 50% are displayed). Moreover, a non negligible number of keypoints are uncorrectly matched by SIFT and ORB. Self-attention and cross-attention layers allow LoFTR algorithm to detect a higher number of keypoints and to individuate strong matches, even in areas where texture is poor.

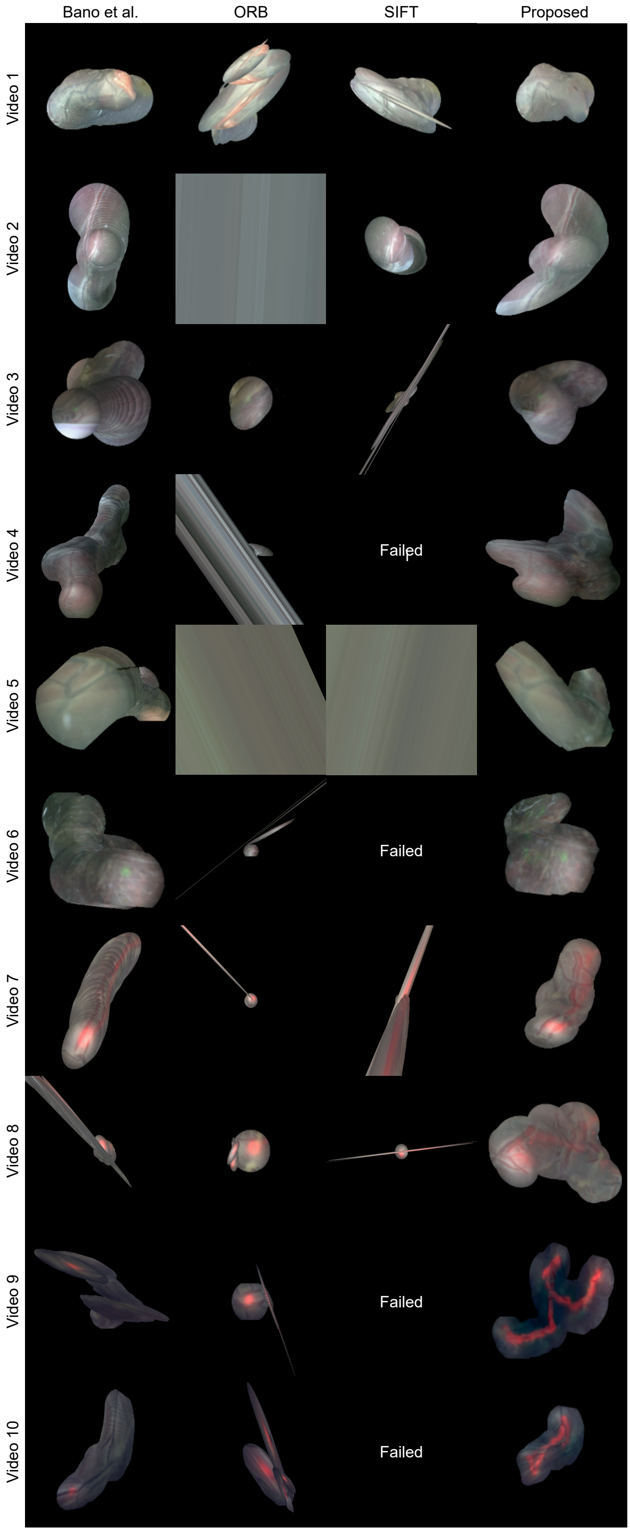

Figure 3: Mosaicking examples for the entire dataset for Bano et al. (***EM1***), SIFT (***EM2***), ORB (***EM3***) and the proposed method.

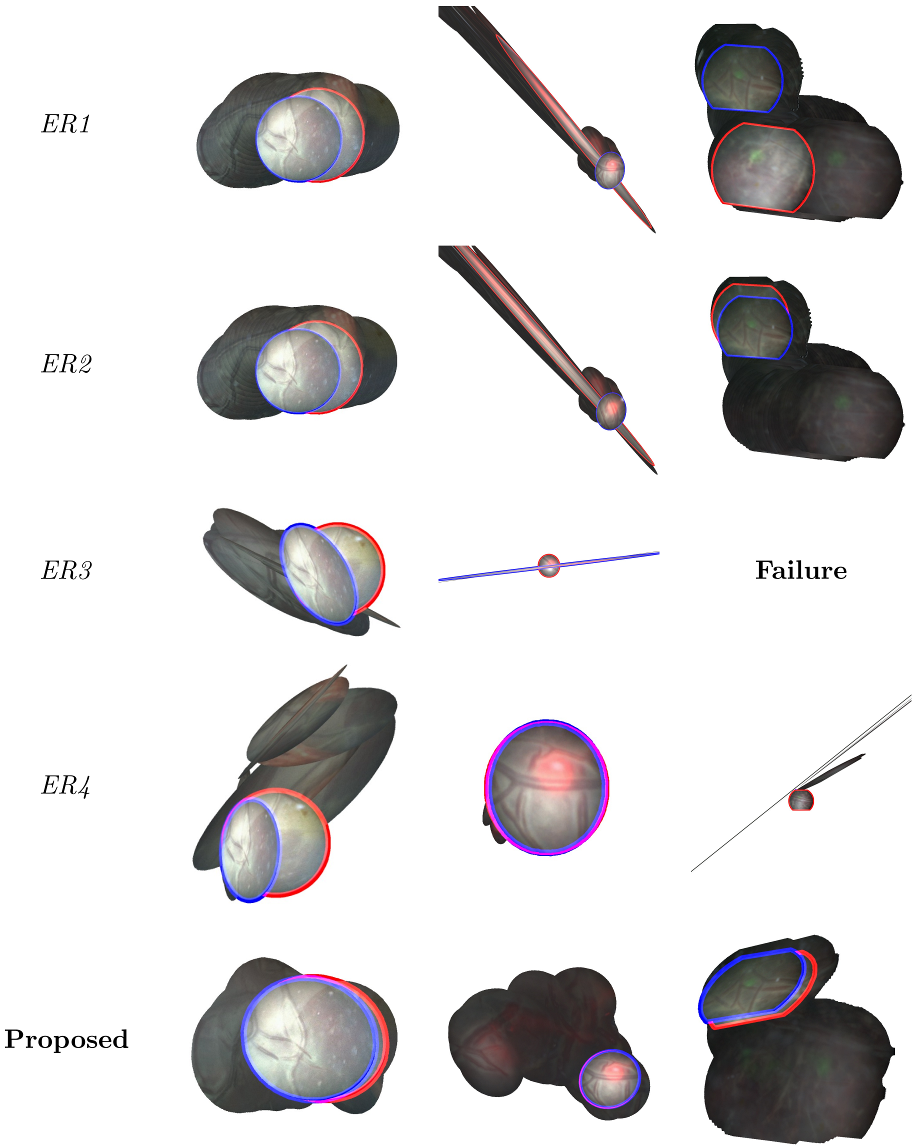

Figure 4: Recovery examples from three different dataset videos for VGG (***ER1***), ResNet50 (***ER2***), SIFT (***ER3***), ORB (***ER4***) and the proposed method. Frame circled in blue is the frame to relocalize, frame circled in red is the nearest keyframe.
